# GSNOR regulates ganoderic acid content in *Ganoderma lucidum* under heat stress through S-nitrosylation of catalase

**DOI:** 10.1038/s42003-021-02988-0

**Published:** 2022-01-11

**Authors:** Rui Liu, Ting Zhu, Xin Chen, Zi Wang, Zhengyan Yang, Ang Ren, Liang Shi, Hanshou Yu, Mingwen Zhao

**Affiliations:** Key Laboratory of Agricultural Environmental Microbiology, Ministry of Agriculture; Microbiology Department, College of Life Sciences, Nanjing Agricultural University, Nanjing, People’s Republic of China

**Keywords:** Environmental microbiology, Fungal physiology

## Abstract

As a master regulator of the balance between NO signaling and protein S-nitrosylation, S-nitrosoglutathione (GSNO) reductase (GSNOR) is involved in various developmental processes and stress responses. However, the proteins and specific sites that can be S-nitrosylated, especially in microorganisms, and the physiological functions of S-nitrosylated proteins remain unclear. Herein, we show that the ganoderic acid (GA) content in GSNOR-silenced (GSNORi) strains is significantly lower (by 25%) than in wild type (WT) under heat stress (HS). Additionally, silencing GSNOR results in an 80% increase in catalase (CAT) activity, which consequently decreases GA accumulation via inhibition of ROS signaling. The mechanism of GSNOR-mediated control of CAT activity may be via protein S-nitrosylation. In support of this possibility, we show that CAT is S-nitrosylated (as shown via recombinant protein in vitro and via GSNORi strains in vivo). Additionally, Cys (cysteine) 401, Cys642 and Cys653 in CAT are S-nitrosylation sites (assayed via mass spectrometry analysis), and Cys401 may play a pivotal role in CAT activity. These findings indicate a mechanism by which GSNOR responds to stress and regulates secondary metabolite content through protein S-nitrosylation. Our results also define a new S-nitrosylation site and the function of an S-nitrosylated protein regulated by GSNOR in microorganisms.

## Abstract (web summary)

Liu et al. identify catalase as a target of S-nitrosylation by GSNOR and the specific sites of S-nitrosylation critical for its role in regulating secondary metabolite production in Ganoderma lucidum under heat stress. This study suggests that GSNOR regulates other metabolic pathways in microorganisms through S-nitrosylation of target proteins in response to environmental changes.

## Introduction

A common and convergent feature of numerous biological processes is the production of redox molecules, including reactive oxygen species (ROS) and reactive nitrogen species (RNS), including nitric oxide (NO) and its derivatives, such as S-nitrosothiols (SNOs), peroxynitrite (ONOO^−^), and higher nitrogen oxides (NO*x*)^1^. NO acts as a transcellular messenger molecule involved in a wide range of physiological functions such as growth, development, stomatal closure, and response to biotic or abiotic stress^2–4^. NO and its derivatives can directly modify target proteins by posttranslational modification. NO radical can interact with metalloproteins, termed metal nitrosylation^5^. The modification of tyrosine residues of proteins by ONOO– is termed tyrosine nitration^6^. The formation of an SNO group on cysteine residues of target proteins is called S-nitrosylation^7^. A major regulatory effect of NO is executed through S-nitrosylation via the covalent addition of an NO group onto a cysteine residue to form SNO^8^. The dynamic processes of protein SNOs include denitrosylation by glutathione (GSH) and the formation of S-nitrosogluthionine (GSNO)^9^. GSNO is a natural NO donor and a major bioactive NO species that participates in protein S-nitrosylation. The degree of S-nitrosylation is balanced and regulated by GSNO reductase (GSNOR)^10^. GSNOR, a member of the class III alcohol dehydrogenase family and a GSH-dependent formaldehyde dehydrogenase, is highly conserved in various species^11^. It was subsequently demonstrated that the enzyme has a high affinity for GSNO and determines the equilibrium between S-nitrosylated proteins and GSNO. Therefore, GSNOR has recently received attention for its role in indirectly regulating NO signaling cascades associated with protein S-nitrosylation. S-nitrosylation, as a redox-based posttranslational protein modification, may be integral to NO function in a variety of cellular processes. Similar to other posttranslational modifications, S-nitrosylation has important roles in the regulation of protein activity, subcellular localization, and protein–protein interactions across myriad physiological processes^12^.

GSNOR plays a crucial role in a large array of physiological and pathological processes by regulating protein S-nitrosylation^7^. In mammals, GSNOR-mediated protein S-nitrosylation is necessary for immune function, inflammation, development, and cancer progression^13^. It has been demonstrated that mesenchymal stem cells from GSNOR-deficient mice exhibit decreased adipogenesis and increased osteoblastogenesis caused by the S-nitrosylation of PPARγ^14^. Deficiency of GSNOR results in increases in the S-nitrosylation of dynamin-related protein 1, contributes to mitochondrial dysfunction, and triggers dysregulation of mitochondrial dynamics^15^. GSNOR-KO cell-selective S-nitrosylation of the mitochondrial chaperone TNF receptor-associated protein 1 mediates its sensitization to inhibitors of succinate dehydrogenase in hepatocellular carcinoma^16^. In plants, GSNOR has been shown to regulate growth, development, shoot branching, hypocotyl growth, and responses to various biotic and abiotic stresses^17^. Plants with GSNOR dysfunction are insensitive to ABA-induced stomatal closure by the S-nitrosylation-inhibiting open stomata 1 protein^18^. GSNOR knockout negatively regulates cytokinin signaling by inhibiting histidine phosphotransfer protein activity through S-nitrosylation in *Arabidopsis*^19^. In *Arabidopsis*, S-nitrosylation of the GSNOR-regulated key transcription factor vascular-related nac-domain7 led to the failure of differentiation of ectopic xylem vessel cells^20^. Although S-nitrosylation cysteine sites modified by GSNOR have been frequently reported in plant and mammalian cells, a proteomic approach was designed for the identification of potential S-nitrosylation sites in several proteins. However, few S-nitrosylation sites on proteins have been directly identified experimentally, significantly limiting further understanding of the physiological function of GSNOR. Unambiguous identification of modified cysteine residues is crucial for understanding the mechanism by which GSNOR modulates protein S-nitrosylation. Compared with research on animals and plants, there is also a lack of research evaluating the corresponding information in microorganisms, and this research remains in the initial stage. The GSNOR recombinant protein was expressed, and the biochemical properties were studied in the filamentous fungus *Aspergillus nidulans*^21^. In *Cryptococcus neoformans*, GSNOR abolished GSNO metabolic activity and attenuated virulence^22^. The relevant research reflects the tentative exploration of GSNOR activity in fungi, but the mechanism by which GSNOR regulates physiological processes through S-nitrosylation needs to be addressed in depth. Identification and determination of S-nitrosylated target sites and proteins will help reveal the role of S-nitrosylation regulation by GSNOR in the control of development and responses to environmental changes.

*Ganoderma lucidum* is a well-known medicinal mushroom contributing to numerous biological activities, including anticancer, immunomodulator, antioxidant, hypoglycemic, and cardioprotective activities^23–25^. Ganoderic acid (GA) represents one of the most abundant secondary metabolites in *G. lucidum* and has attracted extensive research interest. In a previous study, we found that multiple environmental factors and signal molecules participate in the regulation of GA content^26^. Currently, there are few studies on the mechanism by which environmental factors regulate secondary metabolite content in higher basidiomycetes, and *G. lucidum* is an ideal model for further investigation. However, the effect of GSNOR, as a stress response factor, on the process of secondary metabolite accumulation under heat stress (HS) conditions have rarely been addressed. There is little research on GSNOR-mediated S-nitrosylated proteins and the specific targets of these proteins in fungi. In this study, we combined physiological and genetic experiments to investigate the mechanisms by which GSNOR regulates GA content via protein S-nitrosylation under HS conditions. In this study, we identified the targets for S-nitrosylation and the regulation of protein function by S-nitrosylation in microorganisms. In addition, the results also showed the importance of GSNOR-mediated protein S-nitrosylation in HS signaling pathway responses.

## Results

### GSNOR silencing inhibits GA accumulation induced by HS in *G. lucidum*

**Fig. 1:**
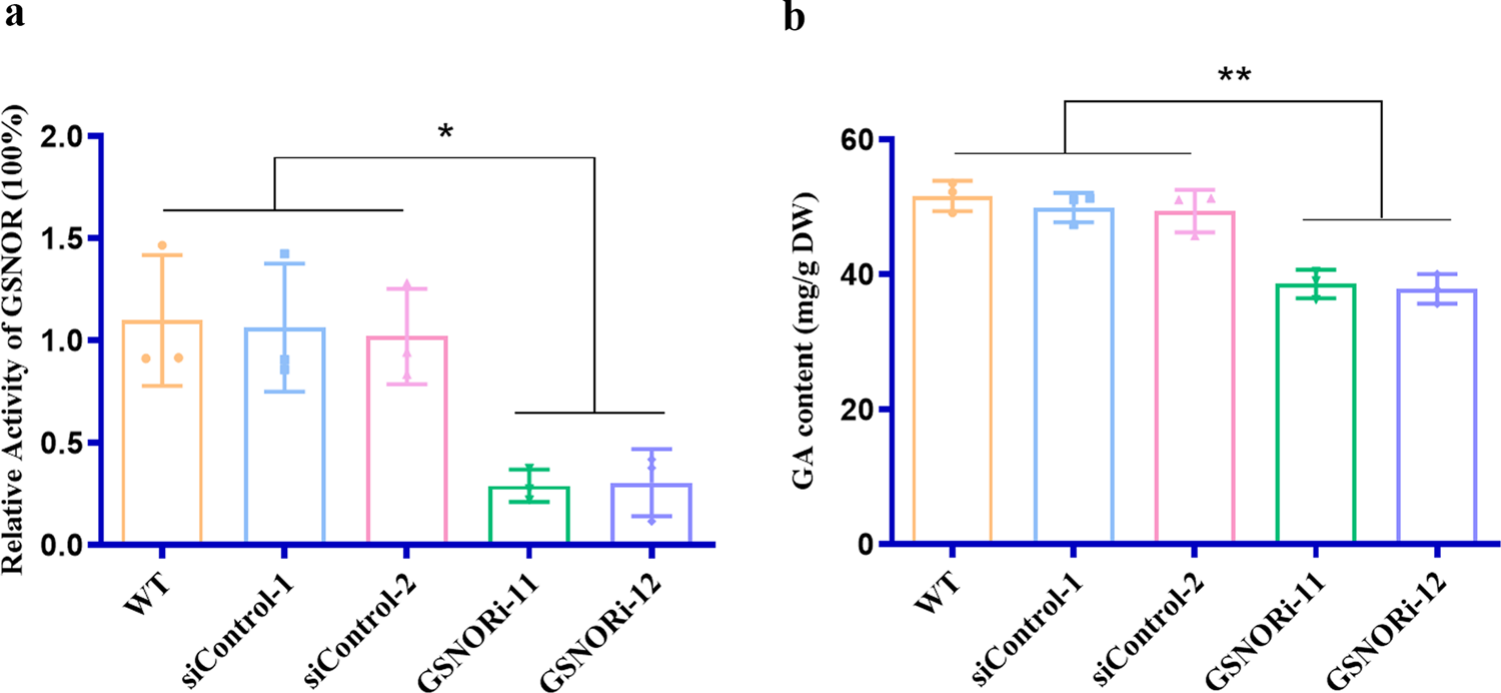
GSNOR activity and GA content in the WT and GSNOR-silenced strains. **a** Protein extracts were prepared from WT and GSNORi mycelia samples, and GSNOR activity was measured as the consumption of NADH in the supernatants via absorbance at 340 nm. **b** The WT strains and GSNOR-silenced strains were grown for 5 days in PDA liquid cultures with shaking and then exposed to 42 °C for 12 h and shifted to 28 °C until the 7th day in stationary. The GA contents were measured in the heat-stressed strains. Data are presented as the mean ± SD based on three independent experiments (*P < 0.05, **P < 0.01 by one-way ANOVA).

The *Dichomitus squalens* (GenBank accession no. XP_007364207.1) GSNOR gene was subjected to a BLAST search against the *G. lucidum* genome database (*G. lucidum* strain 260125-1, NCBI taxid: 1077286)^27^, and the corresponding *G. lucidum* GSNOR gene (GL29181-R1) was found. To explore the role of GSNOR in GA content, GSNOR-silenced strains were constructed by cloning GSNOR-silenced sequences from cDNA into RNA interference (RNAi) constructs using cassette plasmids in our laboratory (Supplementary Fig. 1a). Through hygromycin screening and real-time reverse transcription PCR (qRT-PCR) analysis, GSNORi-11 and GSNORi-12 were found to exhibit 87% and 85% reductions in GSNOR expression compared with the WT strains and the Si-control-1 and Si-control-2 (the empty vector control) strains (Supplementary Fig. 1b). To further examine the silencing efficiency in the GSNORi-11 and GSNORi-12 strains, GSNOR activity was measured. It was also observed that knockdown in the GSNORi-11 and GSNORi-12 strains resulted in a significant reduction of approximately 70% in GSNOR activity compared with that in the WT, Si-control-1, and Si-control-2 strains (Fig. 1a). Thus, the GSNORi-11 and GSNORi-12 strains were selected for further analyses. In a previous study, we found that the GA content increased significantly after HS treatment^28^. To elucidate the effect of GSNOR on GA content under HS conditions, the GA content was analyzed in the WT and GSNORi strains. The results showed a significantly higher GA content (25%) in the WT strains than in the GSNOR-silenced strains under HS conditions (Fig. 1b). The results showed that silencing GSNOR inhibited GA accumulation induced by HS in *G. lucidum*.

### Effects of GSNOR-regulated ROS homeostasis under HS conditions

**Fig. 2:**
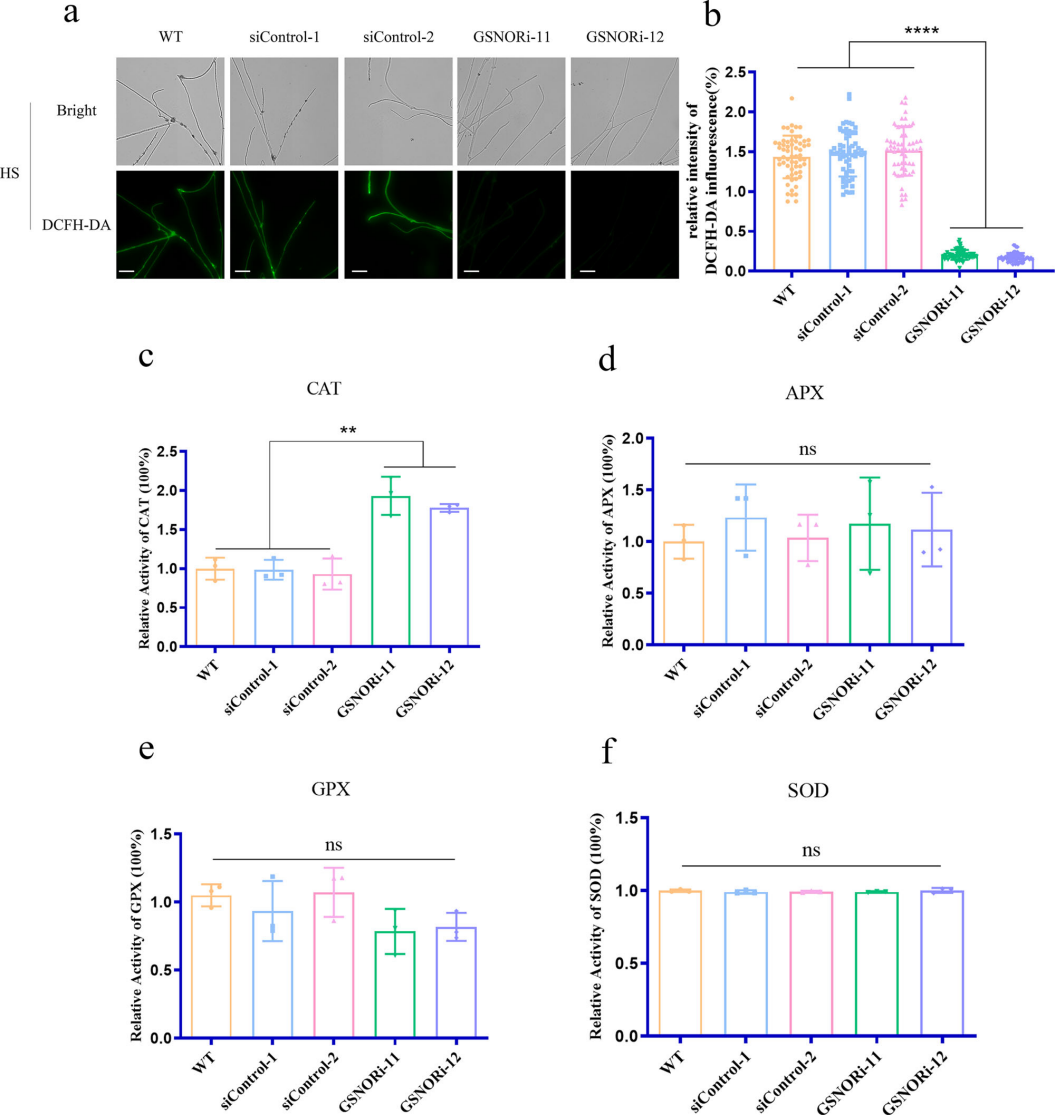
ROS content and major antioxidant enzyme activity in the WT and GSNOR-silenced strains under HS conditions. **a** Coverslips were placed on the bottom of Petri dishes; when the WT and GSNOR-silenced strains cultured on mycelium grew over the coverslips, they were exposed to 42 °C for 20 min. The ROS content was detected by staining with DCFH-DA. Scale bar, 100 μm. **b** Relative ROS content in the WT and GSNOR-silenced strains treated with HS. The WT and GSNOR-silenced strains were cultured for 5 days at 28 °C, then exposed to 42 °C for 20 min. **c**–**f** The CAT (**c**), APX (**d**), GPX (**e**), and SOD (**f**) activity was measured in the WT and GSNOR-silenced strains under HS conditions. Data are presented as the mean ± SD of data from three independent experiments (ns not significant; **P < 0.01, ****P < 0.0001 by one-way ANOVA).

In previous studies, we found that HS-induced ROS increased GA accumulation in *G. lucidum*^29^. GSNOR has emerged as a key player in the control ROS homeostasis under stress conditions. To elucidate the mechanism by which GSNOR inhibits HS-induced GA accumulation, the levels of ROS were determined in WT and GSNOR-silenced strains. In comparison to the WT strain, the GSNOR-silenced strains accumulated approximately 87% lower levels of ROS under HS conditions (Fig. 2a, b). To further test the correlation between GSNOR and ROS, the activities of major antioxidant enzymes, including CAT, glutathione peroxidases (GPX), ascorbate peroxidase (APX), and superoxide dismutase (SOD), were analyzed. The data showed that the GSNOR-silenced strains showed greatly increased CAT activity, and an 80% increase in CAT activity was detected in GSNOR-silenced strains compared with the WT strain under HS conditions (Fig. 2c). However, very similar levels of GPX, APX, and SOD activities were observed in the GSNOR-silenced and WT strains (Fig. 2d–f). This result suggested that the effect of GSNOR on HS-mediated ROS signaling occurred via alterations in CAT activity under HS conditions.

### Reduced ROS accumulation and GA content in GSNORi strains were recovered by inhibition of CAT activity under HS conditions

**Fig. 3:**
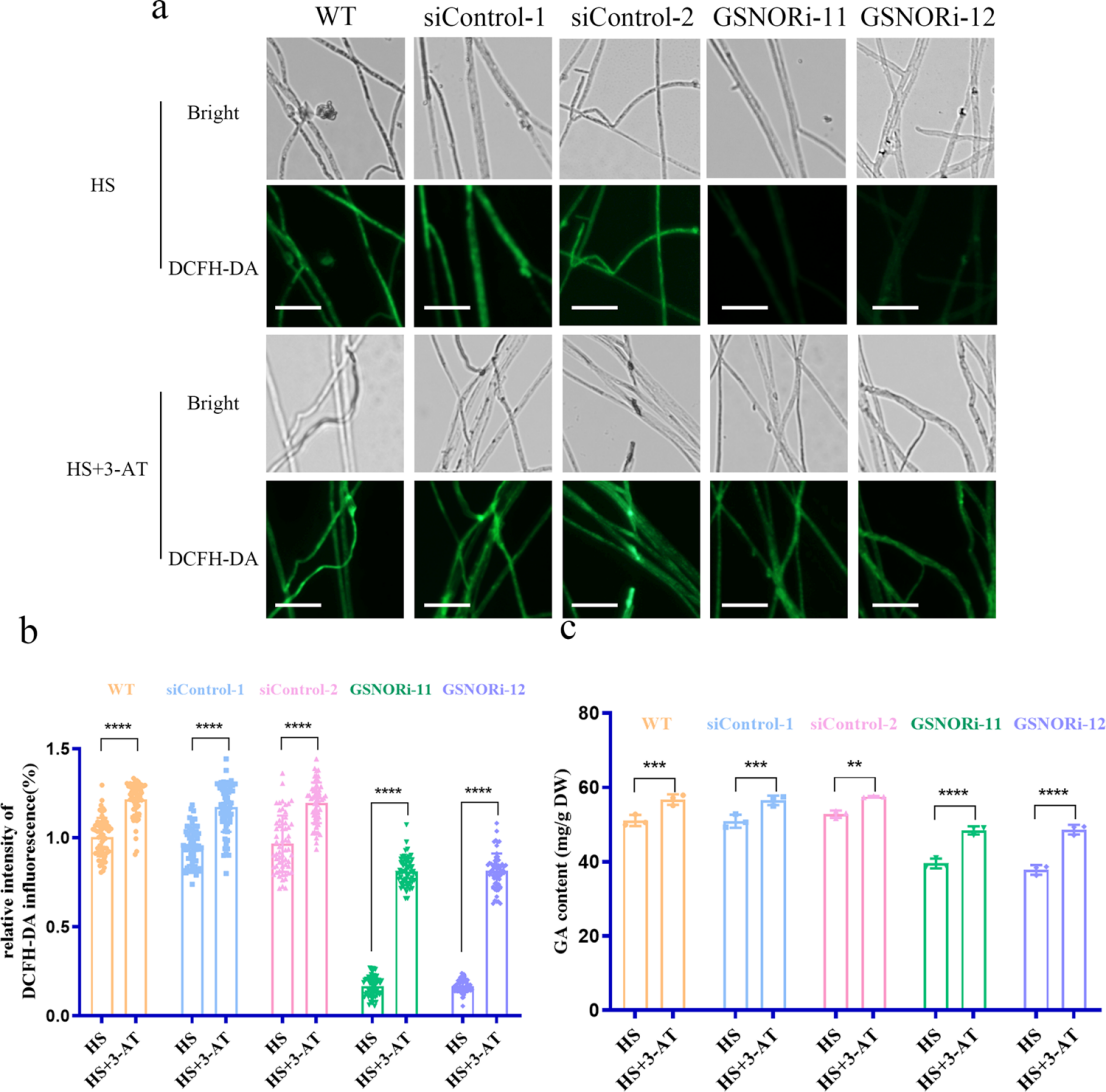
ROS and GA content in the GSNOR-silenced strains treatment with 3-AT under HS conditions. **a** Coverslips were placed on the bottom Petri dishes; after the WT and GSNOR-silenced strains cultured on mycelium grew onto the coverslips, the fungal mycelium was transferred onto a PDA plate with 3-amino-1,2,4-triazole (3-AT) for 30 min, then exposed to 42 °C for 20 min. ROS content was measured by staining with DCFH-DA in the WT and GSNOR-silenced strains untreated or treated with 3-AT. Scale bar, 100 μm. **b** ROS levels in the WT and GSNOR-silenced strains treated with 3-AT. **c** The WT and GSNORi strains were grown for 5 days in PDA liquid cultures with shaking and then treated with 3-AT for 30 min. The strains were exposed to 42 °C for 12 h and shifted to 28 °C until the 7th day in stationary. The GA content in the GSNOR-silenced strains was untreated or treated with 3-AT in the heat-stressed strains. Data are presented as the mean ± SD of data from three independent experiments (**P < 0.01, ***P < 0.001; ****P < 0.0001 by two-way ANOVA).

The silencing of GSNOR increased the CAT activity and decreased the content of GA. Therefore, to determine the role of CAT in GSNOR-mediated regulation of ROS and GA levels under HS conditions, WT and GSNORi strains were treated with 3-amino-1,2,4-triazole (3-AT), an inhibitor of CAT. DCFH-DA analysis showed that the addition of 2 mM 3-AT resulted in a 30% increase in the ROS content compared to that in the untreated WT strain, and the ROS content increased significantly by 84% in the GSNORi strains after treatment with 3-AT compared with the level in the untreated sample (Fig. 3a, b). In addition, the GA level was determined in the WT and GSNOR-silenced strains in the presence of 3-AT. After the addition of 2 mM 3-AT, the GA content was higher (by approximately 6%) than that of in untreated WT strain. However, the accumulation of GA increased significantly (by 27%) after 3-AT treatment in the GSNORi strains under HS conditions (Fig. 3c). The results showed significantly increased GA content after 3-AT treatment compared to that in untreated GSNORi strains. In conclusion, the inhibited CAT activity in the GSNOR-silenced strains caused increased GA content. These results suggested that the silencing of GSNOR inhibited GA accumulation through increased CAT activity.

### HS-induced GA accumulation was reduced in GSNORi strains via CAT modulation

To determine whether the reduced accumulation of ROS in the GSNOR-silenced strains was due to CAT under HS conditions, CAT-silenced strains of *G. lucidum* were constructed by RNAi using cassette plasmids established in our laboratory (Supplementary Fig. 2a)^30^. The efficacy of gene silencing was assessed via qRT-PCR analyses; two CAT-silenced strains (CATi-8 and CATi-14) were used for further analysis, and si-Control-1 and si-Control-2 were selected as empty vector control strains (Supplementary Fig. 2b). CAT activity was markedly decreased by 70% in CATi-8 and CATi-14 compared with that in the WT strain (Supplementary Fig. 2c). Thus, the CATi-8 and CATi-14 strains were selected for further analyses.

**Fig. 4:**
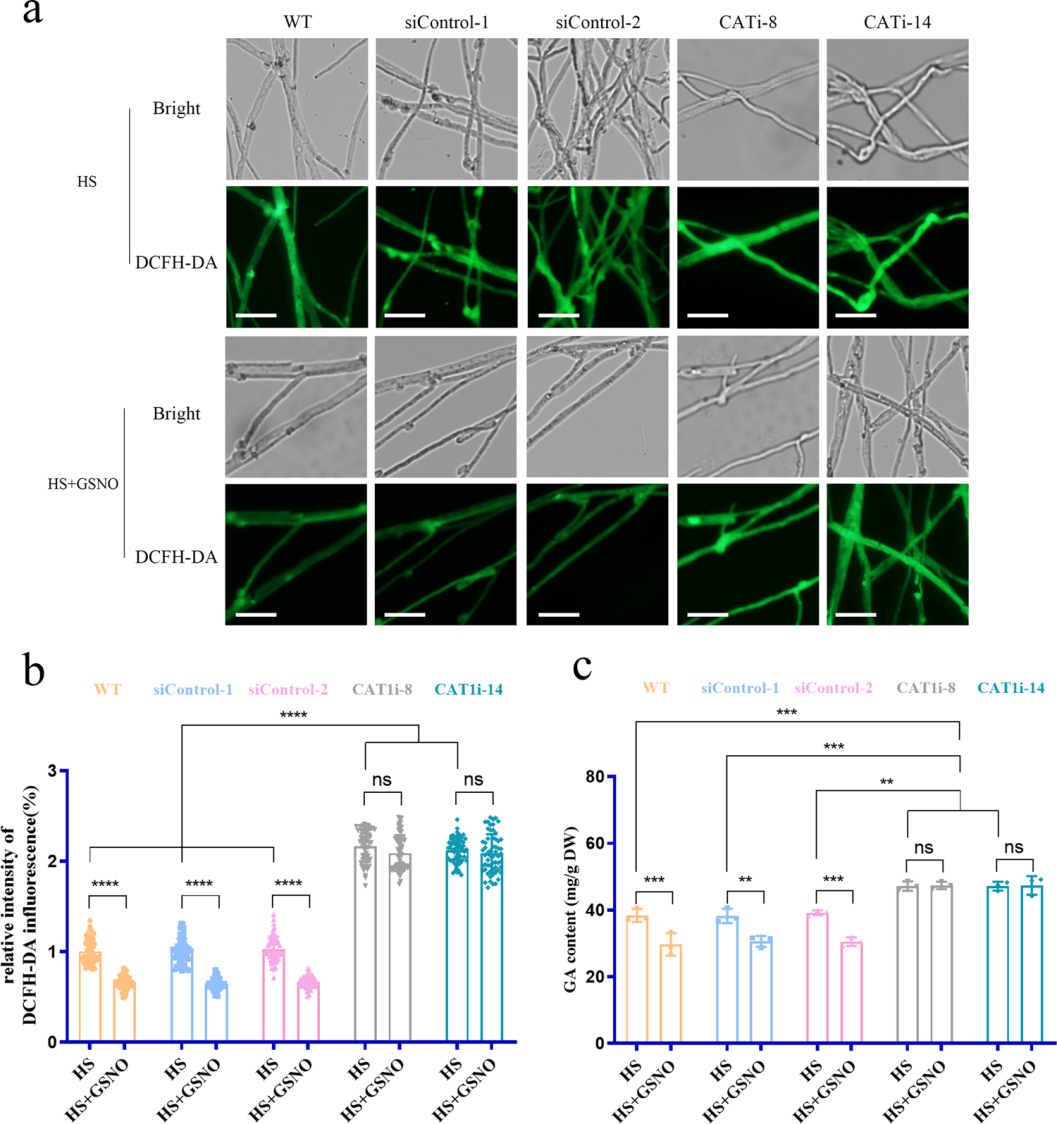
ROS and GA content in the CAT-silenced strains treatment with GSNO under HS conditions. **a** Coverslips were placed on the bottom Petri dishes; after the WT and CAT-silenced strains cultured on mycelium grew onto the coverslips, the fungal mycelium was transferred onto a PDA plate with S-nitrosogluthionine (GSNO) for 30 min, then exposed to 42 °C for 20 min. ROS content was measured by staining with DCFH-DA in the WT and CAT-silenced strains untreated or treated with GSNO. Scale bar, 100 μm. **b** ROS levels in the WT and CAT-silenced strains treated with GSNO. **c** The WT and CATi strains were grown for 5 days in PDA liquid cultures with shaking and then treated with 3-AT for 30 min. The strains were exposed to 42 °C for 12 h and shifted to 28 °C until the 7th day in stationary. The GA content in the CAT-silenced strains was untreated or treated with GSNO in the heat-stressed strains. Data are presented as the mean ± SD of data from three independent experiments (**P < 0.01, ***P < 0.001; ****P < 0.0001 by two-way ANOVA).

To evaluate the effect of CAT on the GSNOR-mediated regulation of ROS accumulation, the ROS levels were also quantified by staining with DCFH-DA in the WT and two CAT-silenced strains. In contrast, silencing CAT significantly increased ROS accumulation by ~115% compared with that in the WT and si-Control strains under HS conditions (Fig. 4a, b). Exogenous GSNO treatment of the WT strains decreased the level of ROS by 36%. In the CAT-silenced strains, the decrease in ROS level after GSNO treatment was undetectable (Fig. 4a, b). These data showed that GSNOR regulated ROS homeostasis, possibly through CAT, under HS conditions.

To further test whether CAT participates in the regulation of GA content by GSNOR under HS conditions, the levels of GA were evaluated in the WT and two CAT-silenced strains in the presence or absence of GSNO, the substrate of GSNOR. The results showed that the levels of GA were significantly higher (22%) in the CAT-silenced strains than in the WT and si-Control strains under HS conditions (Fig. 4c). In the WT strains, the GA content was reduced by 29% upon exogenous application of GSNO compared with that in the untreated WT strain. However, the GSNO-treated CAT-silenced strains did not show any influence on the GA content compared with the untreated silenced strains (Fig. 4c). Together, these results suggested that GSNOR inhibits GA accumulation through CAT under HS conditions. These findings indicated that CAT contributes to decreasing the level of ROS and regulates GA accumulation mediated by GSNOR.

### CAT was S-nitrosylated in vitro and in vivo

**Fig. 5:**
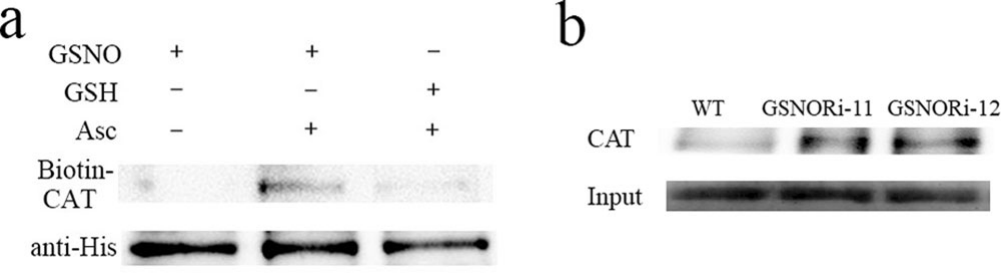
S-nitrosylation of the CAT protein in vitro and in vivo. **a** Recombinant CAT was treated with GSH (negative control for GSNO) or GSNO to measure S-nitrosylation by the biotin switch method. Without sodium ascorbate (Asc) sample as another negative control. Protein samples were separated via SDS–PAGE and analyzed by immunoblotting using an anti-biotin antibody and the His tag antibody. **b** Protein extracts were prepared from WT and GSNOR-silenced strains, and the proteins were analyzed by the biotin-switch assay. The protein was incubated with streptavidin beads, which were then washed to detect the S-nitrosylation of the CAT protein in the WT and GSNOR-silenced strains. Equal loading was verified by antibodies specific for actin and CAT.

Protein S-nitrosylation is a fundamental mechanism for the transduction of GSNOR bioactivity^31^. To verify the mechanism by which CAT activity is regulated by GSNOR under HS conditions’, the S-nitrosylation level of CAT recombinant proteins in vitro was determined using the biotin switch method. The CAT recombinant proteins were labeled with biotin to evaluate S-nitrosylation. As shown in Fig. 5a, CAT was S-nitrosylated, which was induced by GSNO, but S-nitrosylation was not observed in samples treated with GSH (the control for GSNO). The sample treatment without ascorbate sodium (Asc) served as a negative control (Fig. 5a). To further determine whether CAT is S-nitrosylated in vivo, endogenous proteins were extracted from WT strains and GSNOR-silenced strains. The proteins were subjected to biotin switch analysis. Biotin-labeled proteins were purified with streptavidin beads and immunoblotted with an anti-CAT antibody. A low level of S-nitrosylation of CAT could be detected in the WT strains, and a higher level of S-nitrosylation of CAT could be detected in the GSNOR-silenced strains (Fig. 5b). These results suggest that CAT was S-nitrosylated in vitro and in vivo.

To further demonstrate that the potential site is required for GSNOR-mediated S-nitrosylation in the CAT recombinant protein, LC-MS/MS was used to analyze the recombinant CAT protein after GSNO treatment. The CAT protein has 6 cysteine residues, but only three cysteine residues, Cys (cysteine) 401, Cys642, and Cys653, were identified as SNO sites by LC–MS/MS analysis. The mass shift due to the Cys-biotin adduct (+428.19 Da) was present in either the y- or b-ion series (y-ions in blue, b-ions in yellow). The presence of a biotin adducts on Cys401, Cys642, and Cys653 indicated that these Cys residues were S-nitrosylated (Supplementary Fig. 3a–c). These results suggested that Cys401, Cys642 and Cys653 in CAT were S-nitrosylated specifically.

### GSNOR regulates the activity of CAT via S-nitrosylation

**Fig. 6:**
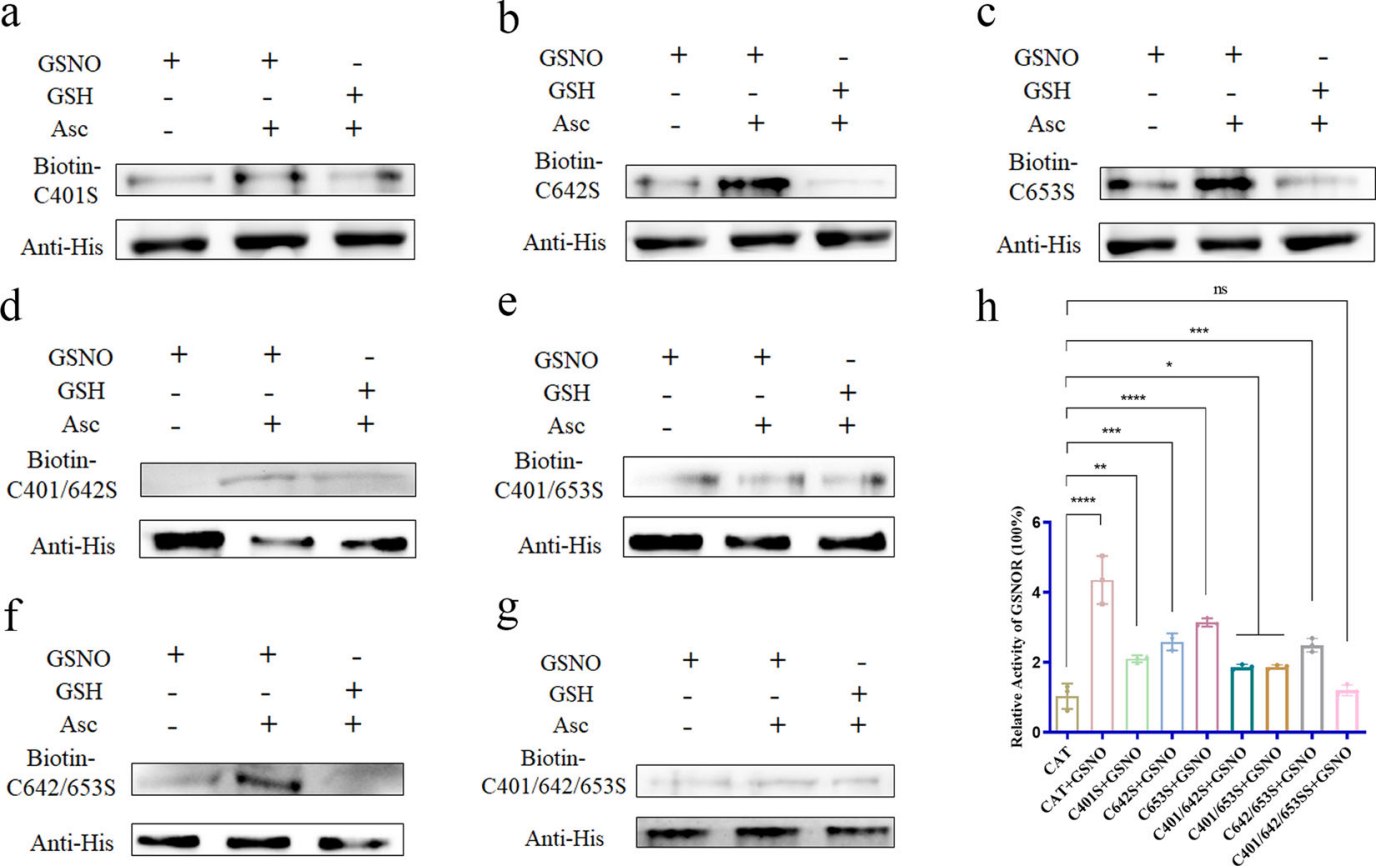
Effects of GSNO treatment on CAT S-nitrosylation and activity. The recombinant CAT protein mutants C401S, C642S, C653S, C401/642S, C401/653S, C642/653S, and C401/642/653S were treated with GSH (negative control for GSNO) and GSNO. Then the protein is subjected to the biotin switch method to determine S-nitrosylation. The samples without Asc as a negative control. Proteins sample were separated via SDS–PAGE and analyzed by immunoblotting using an anti-biotin antibody and the His tag antibody. **a**–**c** C401S, C642S, and C653S are recombinant CAT protein mutants in which the cysteine residues at positions 401, 642, and 653 were changed to serine (Ser). **d**–**f** C401/642S, C401/653S, and C642/653S are recombinant CAT protein mutants in which the cysteine residues at positions 401/642, 401/653, and 642/653 were changed to serine (Ser). **g** C401/642/653S is a recombinant CAT protein mutant in which the cysteine residues at positions 401/642/653 were changed to serine (Ser). **h** The CAT activity of the different CAT proteins was determined as the rate of H_2_O_2_ decomposition per minute by measuring the absorbance at 240 nm with and without GSNO treatment. Data are presented as the mean ± SD of data from three independent experiments (ns not significant; *P < 0.05, **P < 0.01, ***P < 0.001; ****P < 0.0001 by one-way ANOVA).

Since Cys401, Cys642 and Cys653 act as targets for S-nitrosylation in the CAT protein, these S-nitrosylation Cys residues may interfere with CAT activity regulated by GSNOR. Each S-nitrosylated Cys residue was individually changed to serine (Ser) (C401S, C642S, and C653S), which cannot be S-nitrosylated, by site-directed mutagenesis. Furthermore, double and triple Cys-to-Ser mutations were generated at these S-nitrosylation sites (C401/642S, C401/653S, C642/653S, and C401/642/653S) to determine the level of CAT S-nitrosylation. The C401S, C642S, and C653S single mutants (Fig. 6a–c) and C401/642S, C401/653S and C642/653S double mutants (Fig. 6d–f) showed reduced levels of S-nitrosylation, but among the mutated proteins, the triple-mutant protein C401/642/653S had the lowest level of S-nitrosylation (Fig. 6g). In addition, the activity of CAT was further examined in the different CAT proteins after treatment with GSNO. The results showed that GSNO exposure markedly increased the CAT activity of the control CAT protein relative to that of the untreated CAT protein. There were increases to a certain degree in the activities of the C401S, C642S, and C653S single-mutant proteins and C401/642S, C401/653S, and C642/653S double-mutant proteins after GSNO treatment compared with the activity of the untreated protein. Interestingly, among both the single and double mutants, CAT activity was lower if C401 was mutated. In addition, GSNO treatment did not change the CAT activity of the C401/642/653S protein (Fig. 6h). Thus, S-nitrosylation of CAT may increase the activity of this enzyme, and Cys401 may play a pivotal role in CAT activity. According to the above results, GSNOR regulates the activity of CAT via S-nitrosylation. Together, these results suggest that GSNOR, through S-nitrosylation of CAT, influences CAT activity and that Cys401 has a relatively important role in CAT activity.

## Discussion

The mushroom *G. lucidum* has been used for centuries in Asian countries to treat disorders and to promote vitality and longevity because of its supportive effects on the immune system^32^. GA is the main bioactive component in this mushroom, with roles including inhibition of tumor growth and antioxidant, anti-inflammatory, and hypolipidemic activity^33–35^. Accordingly, the GA content is often used as a standard to measure the overall quality of *G. lucidum*. Previous research has shown that multiple signal molecules (such as, ROS, NO, Ca^2+^, H_2_S) impact GA production^28,36–38^. Among them, ROS are reported to play roles that regulate GA production. For example, methyl jasmonate regulates the GA content through ROS signaling generated by NADPH oxidase^39^. Heat stress induced a significant increase in the ROS content, and the ROS increased the GA accumulation^29^. The ornithine decarboxylase-mediated production of putrescine regulates intracellular ROS levels, which increased GA accumulation^40^. However, the lack of intensive research on the regulatory mechanism of GA accumulation limited the development and application of its biological activity. In this study, we found that GSNOR regulated GA accumulation by changing the ROS content. This result is consistent with the findings of previous studies. However, the mechanism of ROS-regulated GA accumulation still needs further study.

To better understand the details of the changes in metabolites regulated by GSNOR, the content of acetyl-CoA (the intermediate converted to GA), pyruvate (the intermediate converted to acetyl-CoA), and *G. lucidum* extracellular polysaccharide (another secondary metabolite in the *G. lucidum*) were detected in the WT and GSNOR-silenced strains under HS conditions. We found that the GA, acetyl-CoA, and pyruvate contents were significantly lower in GSNORi strains than that in the WT strains under HS conditions (Supplementary Fig. 4a, b). However, *G. lucidum* extracellular polysaccharide accumulates significantly more in the GSNORi strains than that in the WT strains under HS conditions (Supplementary Fig. 4c), no significant differences in intracellular polysaccharide concentration between the WT and GSNOR-silenced strains, (Supplementary Fig. 4d). These results showed that the GSNOR not only regulates the accumulation of ganoderic acid and the *G. lucidum* extracellular polysaccharide but also may be changed the metabolic flux under HS conditions. In *Nicotiana attenuate*, GSNOR is also required for regulating the accumulation of secondary metabolites caffeoyl putrescine and diterpene glycosides in present methyl jasmonate. But the specific molecular mechanism is not clear^41^. In the present study, these results suggest that GSNOR regulates GA accumulation via scavenging ROS induced by HS conditions. The results contribute to providing a reference for the understanding of the mechanism on GSNOR regulated the biosynthesis of the secondary metabolite in the other species.

Protein S-nitrosylation, a posttranslational modification, is an important regulatory mechanism by which GSNO reductase (GSNOR) affects target protein function. In recent years, research on target proteins regulated by S-nitrosylation has received much attention because of the importance of protein S-nitrosylation in a broad spectrum of cellular processes^42^. Due to the complexity of predicting the S-nitrosylation sites in proteins and the indirect nature of the existing methods to detect S-nitrosylated proteins, research on the identification of the target sites in S-nitrosylated proteins is still in the preliminary stage. Although a growing number of S-nitrosylated proteins have been revealed^18,43^, these proteins need to be further characterized by specific approaches to determine their physiological relevance. To date, with the development of new techniques, a number of S-nitrosylation sites have been identified from proteome-wide analyses. This study provides a potential aim for future functional studies to identify the targets regulated by GSNOR. However, the information about S-nitrosylation sites and the effects of S-nitrosylation sites on protein function needs to be validated. Therefore, identifying the biological functions of individual S-nitrosylation sites contributes to the understanding of the molecular mechanism underlying the essential role of S-nitrosylation sites in regulating a number of biological processes.

ROS, as signaling molecules, regulate several important physiological responses, and GSNOR has emerged as a key enzyme in the homeostasis of ROS signaling^44^. Moreover, GSNOR functions mainly through the regulation of NO homeostasis and interaction with ROS, thereby modulating oxidative signaling or oxidative stress. The levels of ROS have been confirmed in plants with knockout and overexpression of GSNOR, providing the most compelling evidence for the role of GSNOR as a potential master regulator of ROS levels. For example, the application of the GSNOR inhibitor N6022 significantly increased ROS levels under salinity and cadmium stress in *Solanum habrochaites*^45^. Fe deficiency induced GSNOR expression in tomato, and GSNOR overexpression lines showed significantly decreased accumulation of ROS compared with WT lines after 10 days of Fe deficiency treatment to alleviate Fe deficiency-related damage^46^. A growing number of proteins have been described as targets of posttranslational modifications by GSNOR-mediated S-nitrosylation under stress conditions. Cysteine-based S-nitrosylation might be a general posttranslational protein modification to control ROS homeostasis. In *Arabidopsis*, S-nitrosylation of NADPH oxidase at Cys890 in GSNOR mutants with high concentrations of S-nitrosothiols abolished the ability to synthesize ROS and limited cell death during the hypersensitive response^47^. A higher level of APX1 protein S-nitrosylated at Cys32 was found in *gsnor* allelic mutants compared with WT plants, and S-nitrosylated APX1 protein showed enhanced enzymatic activity for ROS breakdown, leading to increased resistance to oxidative stress^48^. In the current study, we found that silencing GSNOR promoted CAT S-nitrosylation at Cys401, Cys642, and Cys653 and increased CAT activity for scavenging ROS induced by HS. This is the report of CAT, as a S-nitrosylation target protein, being regulated by GSNOR in microorganisms. The results further support the notion that GSNOR-mediated S-nitrosylation may play an important role in the regulation of redox homeostasis-related enzymatic activity for coping with various stresses. These results also demonstrate that the S-nitrosylated sites may differ under different conditions or in different species. This aspect indicates the diversity and richness of S-nitrosylation.

GSNOR is now known to be a critical endogenous regulator in a variety of physiological processes, and this protein functions mainly through the regulation of protein S-nitrosylation. More interestingly, in various processes, there might be different sites of S-nitrosylation in the same protein regulated by GSNOR. For example, GSNO triggers inhibition of GAPDH activity through S-nitrosylation of Cys149 in *Arabidopsis thaliana*^49^. GAPDH was shown to be nitrosylated at Cys155 and Cys159, and these cysteine modifications inactivated the enzymes from *Arabidopsis thaliana*^50^. In addition, S-nitrosylation at different sites may have different effects on protein function. Two cysteine residues within TIR1, namely, C140 and C480, act as putative targets of S-nitrosylation. A C140 single mutant of TIR1 completely abolished the TIR1-AuxIAA interaction, and the interaction was strongly impaired in the C480 single mutant of TIR1. These results indicated that the TIR1-Aux/IAA interaction is dependent on C140 and, to a lesser extent, on C480^51^. TGA1 has four Cys residues (Cys172/Cys260/Cys266/Cys287) that are S-nitrosylated after treatment with 500 μM GSNO. The TGA1 proteins as well as the C260S/C266S double mutants bound the activation sequence-1 element with higher activity after GSNO treatment, and binding could not be observed in the C172S/C287S and C172S/C260S/C266S/C287S quadruple mutants. The results suggest that different S-nitrosylation sites influenced the DNA binding activity of TGA1 to the sequence-1 element^52^. In the present study, we found that Cys401 had a significantly stronger impact on the CAT activity than Cys642 and Cys653 in response to HS in *G. lucidum*. Our results are similar to those of previous studies, and all of these results confirmed that the function of proteins often changes greatly due to different sites being S-nitrosylated. These results suggest that further research is needed to directly identify the biological roles of S-nitrosylation sites and analyze the different impacts of different S-nitrosylation sites on protein function.

GSNOR-regulated redox homeostasis via S-nitrosylation plays a key role in the response to biotic stresses, but it remains unclear how GSNOR modulates its targets under various biotic stresses. Our data suggest that silencing GSNOR inhibited HS-induced ROS accumulation by S-nitrosylation of the major antioxidant enzyme CAT in vitro and in vivo. S-nitrosylation at three sites, namely, Cys401, Cys642 and Cys653, in the CAT protein significantly increased CAT activity. Further research found that Cys401 in the CAT protein plays a relatively important role in curbing excessive ROS and GA accumulation induced by HS. These data establish a molecular framework for GSNOR function during the control of ROS homeostasis. In future work, we will identify a series of S-nitrosylated proteins, which may help to reveal the role of S-nitrosylation in the response to stress in microorganisms. Moreover, identification of modified proteins and specific sites could provide a more accurate description of the underlying mechanism and is crucial for understanding the regulatory effects of S-nitrosylation.

## Methods

### Strains and culture conditions

The *G. lucidum* strain ACCC53264 was provided by the Agricultural Culture Collection of China was used as the wild-type (WT) strain. The strain was grown at 28 °C in potato dextrose agar medium for 7 days. *G. lucidum* mycelium were inoculated on a PDA (potato dextrose agar) plate overlaid with cellophane for 5 days at 28 °C. Then, the strains were shifted to 42 °C for 20 min to visualize ROS. *G. lucidum* strains PDB (potato dextrose broth) liquid cultures were placed at 28 °C with shaking for 5 days and then held stationary and shifted to 42 °C for 12 h until the 7th day to evaluate GA content^28^.

### Construction of RNAi strains

The coding regions of the GSNOR gene and catalase (CAT) gene were using *G. lucidum* cDNA as the template, PCR was performed with the primers GSNORi-F (ACTGGGTACCGCTGGAAAGCCCATTACG), GSNORi-R (ACTGACTAGTGACGGAGACATCTGCGAC), CATi-F (ACTGGGTACCCTCGTGGAAGACCAGATT), and CATi-R (ACTGACTAGTGAGTCAAGGCAGCAAAA) for constructed the fungal RNAi vector^30^. The RNAi-silenced vectors pAN7-dual-GSNORi and pAN7-dual-CATi were transferred into *G. lucidum* by electroporation. The two independent strains silenced with the greatest efficiency were finally selected for subsequent experiments.

### ROS detection assay

The strains were inoculated on a PDA plate overlaid with cellophane for 5 days at 28 °C. The mycelium was then transferred onto a PDA plate with S-nitrosogluthionine (GSNO) and 3-amino-1,2,4-triazole (3-AT) for 30 min. Then, the strains were exposed to 42 °C for 20 min^28^. The samples were stained with 20,70-dichlorodihydrofluorescein diacetate (DCFH-DA) for 20 min to visualize ROS^36^. Fluorescence images were obtained by using a Zeiss Axio Imager A1 fluorescence microscope, and the average fluorescence intensities were measured using ZEN lite software (Zeiss).

### Measurement of enzymatic activity

To measure the enzymatic activity, the mycelia were cultured on a PDA plate overlaid with cellophane. The strains were frozen and ground in liquid N_2_ after exposure to 42 °C for 20 min. The frozen sample (0.3 g) were homogenized in potassium phosphate buffer (50 mM, pH 7.0). The supernatants were centrifuged at 10,000 × *g* for 20 min at 4 °C, and the supernatant obtained following centrifugation was used in enzyme activity assays. CAT activity was determined as the rate of H_2_O_2_ decomposition per minute by measuring the absorbance at 240 nm^53^. SOD activity was assayed is based on the inhibition of the photochemical reduction of nitro blue tetrazolium (NBT)^54^. APX catalyzes the oxidation of ascorbic acid by H_2_O_2_, and APX activity was spectrophotometrically measured at 290 nm and assessed by calculating the rate of ascorbic acid oxidation through the APX Assay Kit (Nanjing Jiancheng Bioengineering Institute). The activity of GPX was measured using the GPX Assay Kit (Beyotime Institute of Biotechnology) that determines the coupled oxidation of NADPH during the glutathione reductase recycling of oxidized glutathione from the GPX-mediated reduction of t-butyl peroxide. GSNOR activity was measured using the GSNO-dependent consumption of NADH. The frozen mycelia (1 g) were homogenized in reaction buffer (20 mM Tris–HCl, pH 8.0 and 200 µM NADH). The GSNO-dependent consumption of NADH in the supernatants was monitored via the absorbance at 340 nm^55^. Protein concentration used was determined by the Bradford method, using bovine serum albumin as a standard.

### Detection and quantification of GA, extracellular polysaccharide, acetyl-CoA and pyruvate

To detect ganoderic acid (GA), *G. lucidum* strains were grown for 5 days in PDA liquid cultures with shaking and then exposed to 42 °C for 12 h and shifted to 28 °C until the 7th day in stationary. GA was extracted from mycelia and measured^56^. In brief, dried mycelia (50 mg) were saponified in 10% (w/v) KOH-75% (v/v) ethanol solution (2 mL) at 50 °C for 2 h, and the mixture was extracted with 2 mL of hexane for three times. The hexane layer was evaporated to dryness under nitrogen, and the residue was dissolved in 500 μL of acetonitrile for the subsequent ultraperformance liquid chromatography (UPLC) analysis.

The content of acetyl-CoA was detected in the culture of *G. lucidum* using an Acetyl CoA (Ac-CoA) ELISA Kit (Sigma). The *G. lucidum* mycelium were extracted with 1.0 M perchloric acid, and then the sample was centrifuged for 10 min at 10,000 × *g*. The acetyl-CoA concentration was determined by a coupled enzyme assay that produces a fluorometric product proportional to the acetyl-CoA. The *G. lucidum* mycelium was suspended in 3 volumes of 80% ethanol and sonicated at 4 °C. Then, Centrifuge the crushed mycelium at 8000 × *g*, 4 °C for 10 min, and the supernatant was the extract containing pyruvate^57,58^. Determination of the pyruvic acid content by UPLC. After the removal of the mycelia by centrifugation, *G. lucidum* extracellular polysaccharide was precipitated by It is obtained by adding 4 volumes of 95% (v/v) ethanol, stirring vigorously, incubating overnight at 4 °C. The insoluble components were suspended in 1 M NaOH and suspended at 60 °C for 1 h to determine the content of the *G. lucidum* extracellular polysaccharide by the phenol-sulfuric acid method^59,60^.

### Expression and purification of recombinant proteins

CAT sequences from *G. lucidum* were deposited in the GenBank (OK469300). The pColdI-CAT, pColdI-CAT C401S, C642S, C653S, C401/642S, C401/653S, C642/653S and CysC401/642/653S expression vectors were transformed into *Escherichia coli* strain BL21 (DE3), and protein expression was induced with 500 μM isopropyl-beta-d-thiogalactopyranoside (IPTG) and the strains were grown at 16 °C for 12 h. The recombinant proteins were purified using the nickel-nitrilotriacetic acid (Ni-NTA) agarose column (Sangon, C600033).

### In vitro S-nitrosylation assay

The purified proteins were subjected to buffer exchange with HEN buffer (HEPES (250 mM, pH 7.7), 1 mM EDTA) containing 10% (v/v) glycerol. The S-nitrosylation of pColdI-CAT, pColdI-CAT C401S, C642S, C653S, C401/642S, C401/653S, C642/653S, and CysC401/642/653S was detected by the biotin-switch assay^61^. The proteins were incubated in GSNO or GSH at room temperature in the dark for 60 min. The non-nitrosylated free cysteine residues of the proteins were then blocked for 1 h at 55 °C, the samples were precipitated by cold acetone and washed 3 times with 70% acetone. The proteins were resuspended in HEPES buffer (HEPES (250 mM pH 7.7), 4 mM EDTA, 1 mM neocuproine and 1% SDS) and after adding 20 mM Asc and 4 mM biotin-HPDP, proteins were incubated at room temperature for 1 h. A sample not treated with Asc was used as the negative control (Cys-SNO is removed with Asc and replaced with biotin-HPDP). After the reaction with the biotin-HPDP, the proteins sample were separated via SDS-PAGE and analyzed by immunoblotting with anti-biotin and anti-His antibodies.

### In vivo S-nitrosylation assay

Extract protein from about 1 g fresh mycelium of *G. lucidum* by homogenization in 1 mL of ice-cold HEN buffer (HEPES (250 mM, pH 7.7), 1 mM EDTA, 0.1 mM neocuproine) and protease inhibitor. The proteins were analyzed by the biotin-switch assay, and the sample was were incubated overnight at 4 °C with streptavidin beads. Wash the beads four times with washing buffer (HEPES (25 mM, pH 7.7), 600 mM NaCl, 1 mM EDTA, and 0.5% [v/v] Triton X-100)^19^. Eluted proteins were analyzed by immunoblotting with anti-CAT and anti-actin antibodies.

### Mass spectrometric analysis of S-nitrosylated residues

His-CAT protein labeled with biotin-HPDP was excised and subjected to in-gel digestion with trypsin and peptides were analyzed by LC–MS/MS. The MS/MS spectra were searched for peptide identification using the *G. lucidum* protein database. The database search included cysteine biotinylation (428.19 Da), cysteine carbamidomethylation and methionine oxidation^19^.

### Statistics and reproducibility

All data presented in this manuscript are from at least three independent experiments. Error bars indicate standard deviation from the mean from triplicate independent experiments. Differences in mean values between groups were analyzed by one-way or two-way analysis of variance (ANOVA) using GraphPad Prism. For statistical representation, *P < 0.05, **P < 0.01, ***P < 0.001, and ****P < 0.0001 are the levels of statistical significance. NS is not significant.

### Reporting summary

Further information on research design is available in the Nature Research Reporting Summary linked to this article.

## Supplementary information

Supplementary Information

Description of Additional Supplementary Files

Supplementary Data 1

Reporting Summary

## Data Availability

All data generated or analyzed during this study are included in this published article (and its Supplementary Information files) or are available from the corresponding author on reasonable request. The source data underlying the graphs in the figure are shown in Supplementary Data 1. The mass spectrometry proteomics data have been deposited to the ProteomeXchange Consortium via the iProX partner repository with the dataset identifiers PXD029922. Uncropped western blots are in Supplementary Figure 5 and 6.
