# Supplementary material for: GSNOR regulates ganoderic acid content in Ganoderma lucidum under heat stress through S-nitrosylation of catalase
Source: Commun Biol. 2022 Jan 11;5:32. doi: 10.1038/s42003-021-02988-0 (PMC8752759; doi:10.1038/s42003-021-02988-0)
Supplement: Supplementary file 1 — Supplementary Information [file 42003_2021_2988_MOESM1_ESM.pdf]

Supplementary Material for

GSNOR regulates ganoderic acid content in *Ganoderma lucidum* under heat stress through S-nitrosylation of catalase

Rui Liu[1], Ting Zhu[1], Xin Chen[1], Zi Wang[1], Zhengyan Yang[1], Ang Ren[1], Liang Shi[1], Hanshou Yu[1], Mingwen Zhao[1]✉

[1]Key Laboratory of Agricultural Environmental Microbiology, Ministry of Agriculture; Microbiology Department, College of Life Sciences, Nanjing Agricultural University, Nanjing, People′s Republic of China

Corresponding author: Mingwen Zhao

✉email: mwzhao@njau.edu.cn

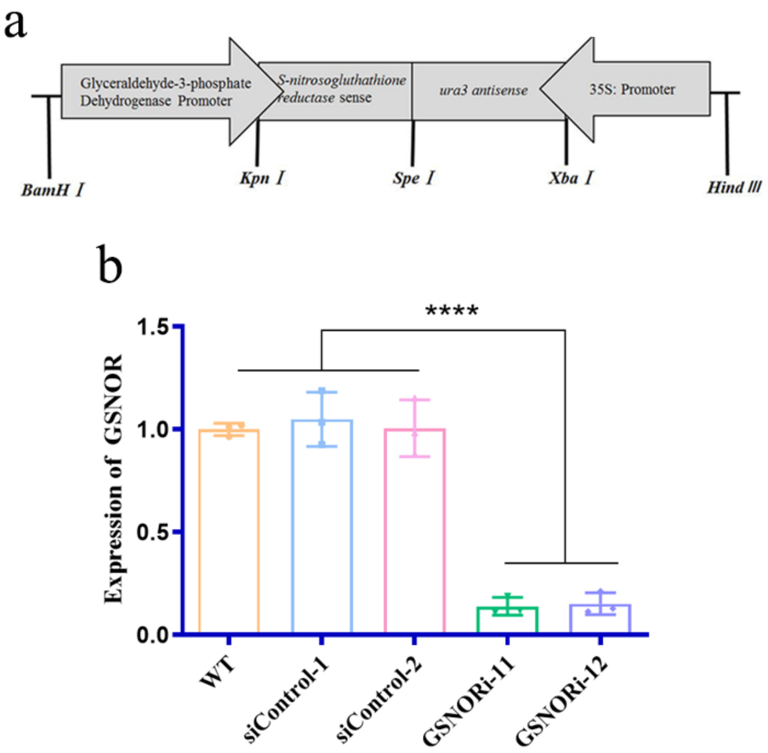

Supplementary Figure 1 Construction of the silenced strains of GSNOR and assessment the silencing efficiency of GSNOR

a. Construction of GSNOR-RNAi expression cassette plasmids. b. The wild-type (WT), siControl-1, siControl-2 (transformed with the empty plasmid), and GSNOR-silenced (GSNORi-11 and GSNORi-12) strains were cultured on PDA plates. The expression levels of *GSNOR* were measured in the candidate strains. Data are presented as the mean ± SD of data from three independent experiments (****$P < 0.0001$ by one-way ANOVA).

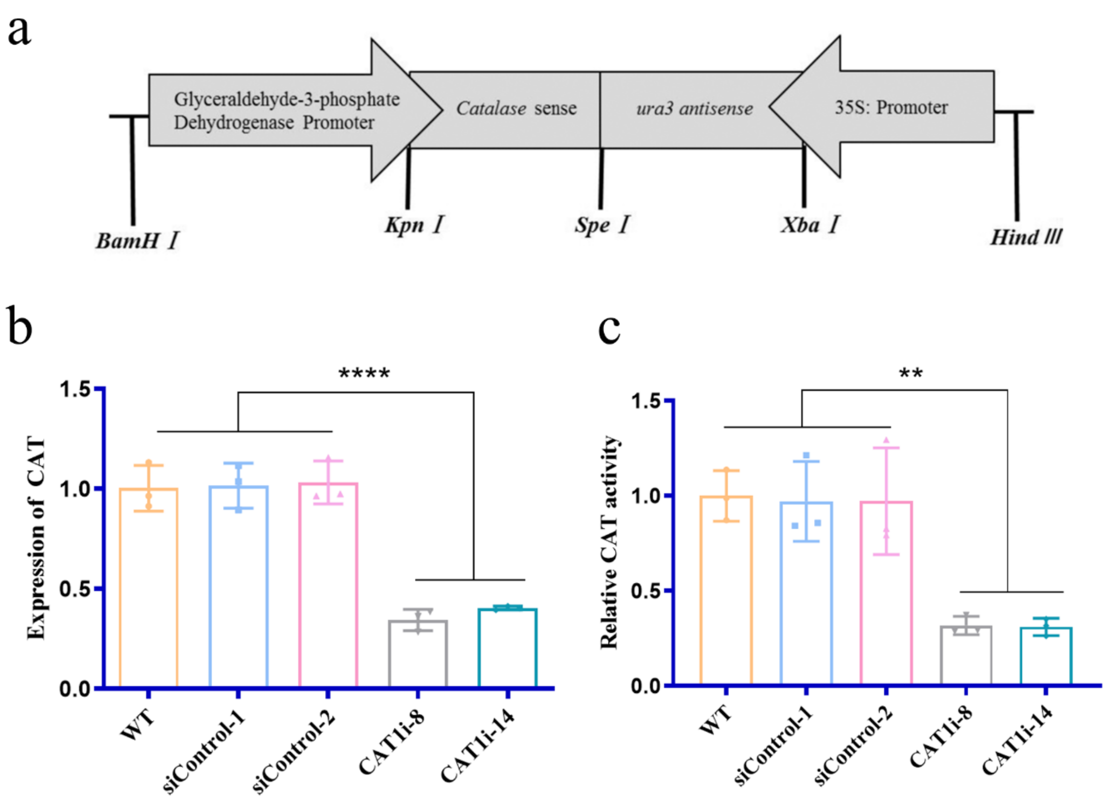

Supplementary Figure 2 Construction of the CAT-silenced strains and assessment of the CAT-silencing efficiency

a. Construction of CAT-RNAi expression cassette plasmids. b. The wild-type (WT), siControl-1, siControl-2 (transformed with the empty plasmid), CAT-silenced (CATi-8 and CATi-14) strains were cultured on PDA plates. The expression levels of the *CAT* in the candidate strains were measured. c. Coverslips were placed on the bottom petri dishes; when the WT and CAT-silenced strains were cultured on mycelium grew on the cover slips. Then CAT activity was determined as the rate of H2O2 decomposition per minute by measuring the absorbance at 240 nm in the CAT-silenced strains. Data are presented as the mean ± SD based on three independent experiments (**$P < 0.01$, ****$P < 0.0001$ by one-way ANOVA).

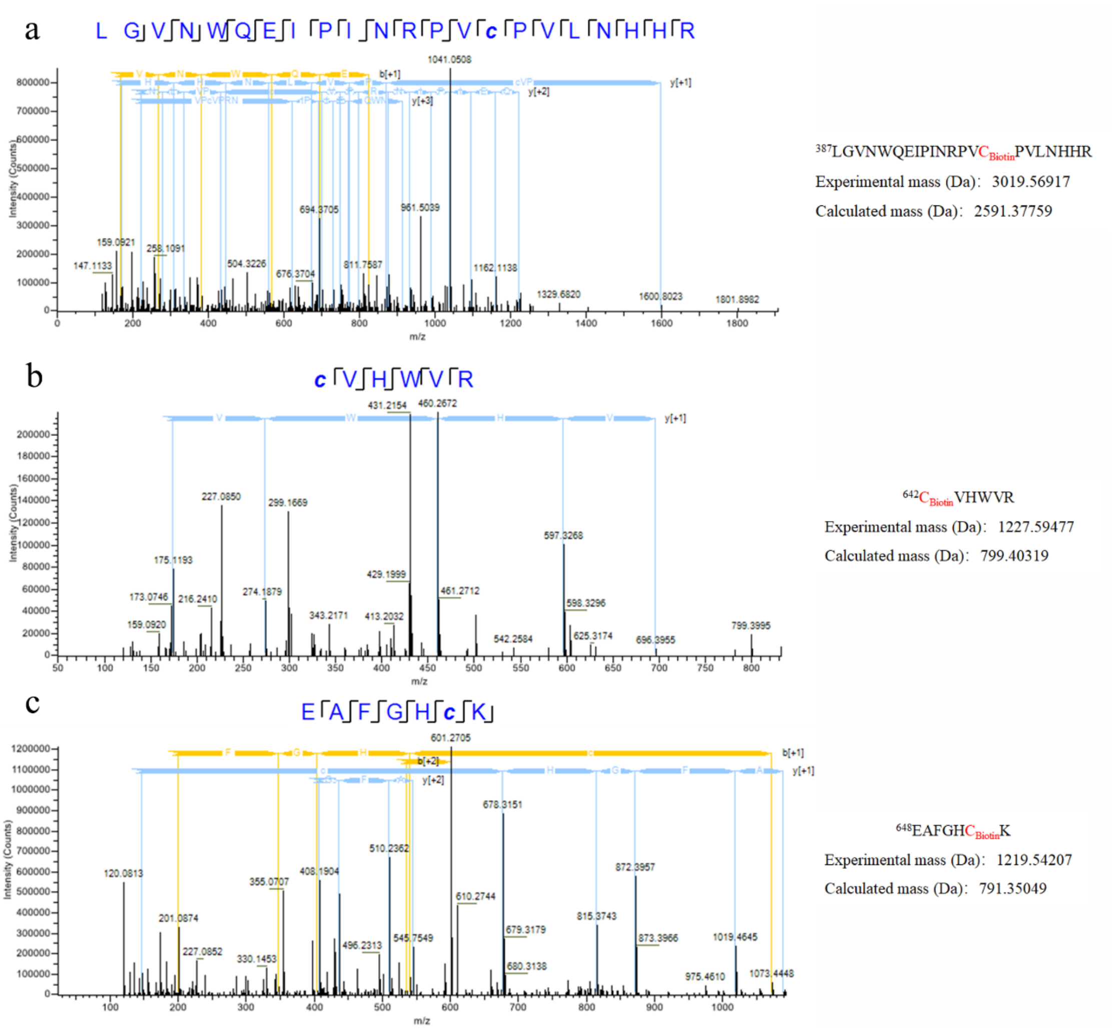

Supplementary Figure 3 The S-nitrosylation sites in recombinant CAT protein were identified by MS as Cys401, Cys642 and Cys653.

a-c. The presence of a biotin adducts on Cys401, Cys642 and Cys653 indicates that these Cys residues were S-nitrosylated. The peptides sequence and the peptides masses were shown above. The mass shift due to the Cys–biotin adduct (+428.19 Da) was present in either the y- or b-ion series (y-ions in blue, b-ions in yellow).

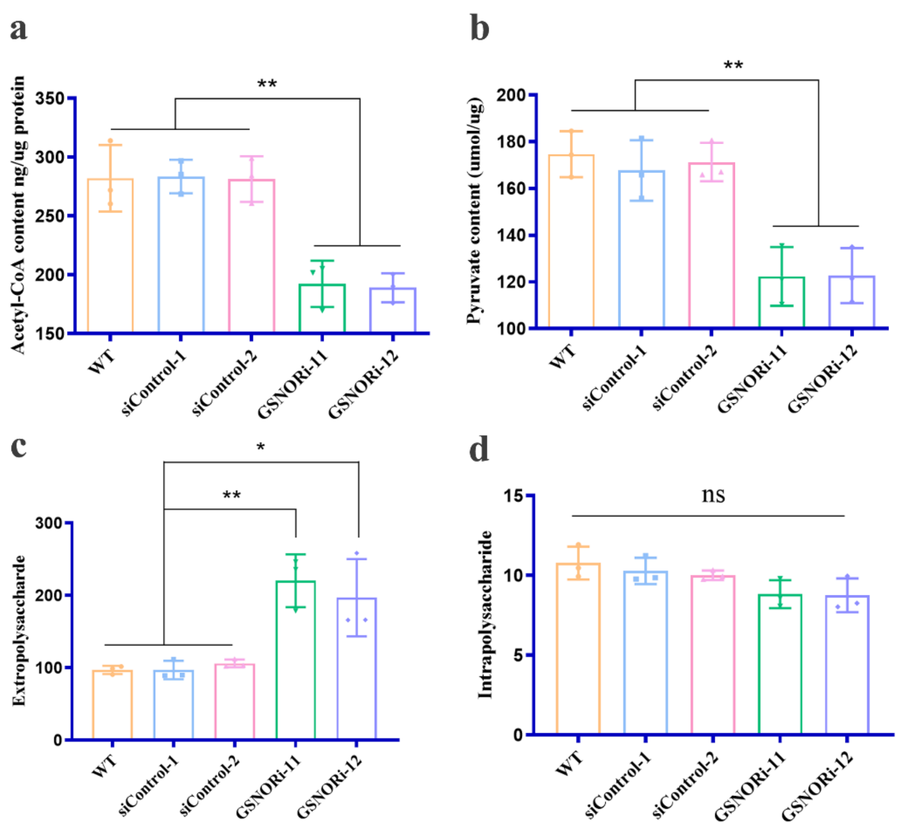

Supplementary Figure 4 The contents of acetyl-CoA, pyruvate and *G. lucidum* polysaccharide in the WT and GSNOR-silenced strains under HS conditions.

a. Acetyl-CoA content in the WT and GSNOR-silenced strains under HS conditions. The acetyl-CoA concentration was determined by a coupled enzyme assay that produces a fluorometric product proportional to the acetyl-CoA. b. The pyruvate content in the WT and GSNOR-silenced strains under HS conditions. The mycelia were suspended in 80% ethanol and sonicated at 4 °C. Then to assay the pyruvate content. c-d. The extracellular polysaccharide and intracellular polysaccharide content in the WT and GSNOR-silenced strains under HS conditions. *G. lucidum* extracellular polysaccharide was were measured using the phenol-sulfuric acid method content of *G. lucidum* extracellular polysaccharide. Data are presented as the mean ± SD based on three independent experiments (ns not significant; $*P < 0.05$, $**P < 0.01$ by one-way ANOVA).

Source data for Figure 5a

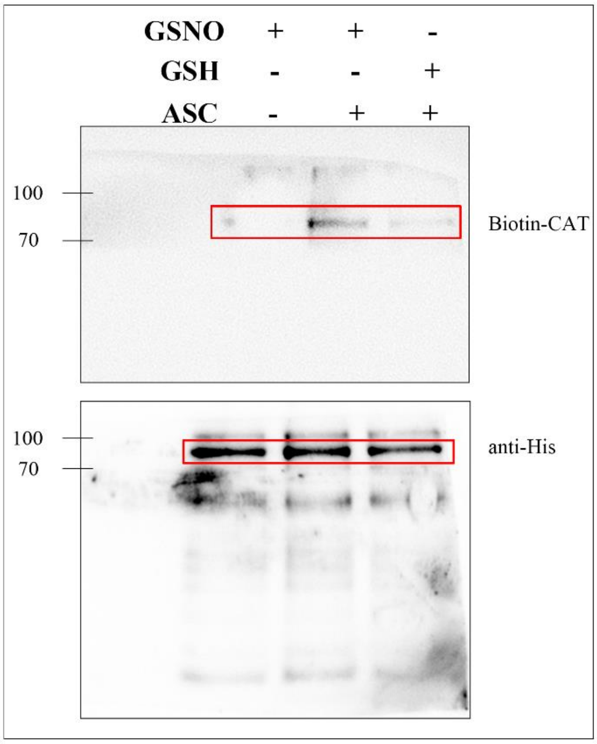

Source data for Figure 5b

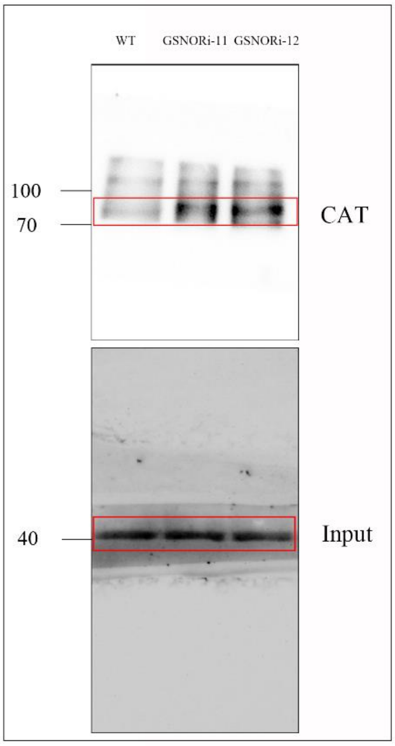

Supplementary Figure 5. Uncropped Western blots.

Source data for Figure 6a

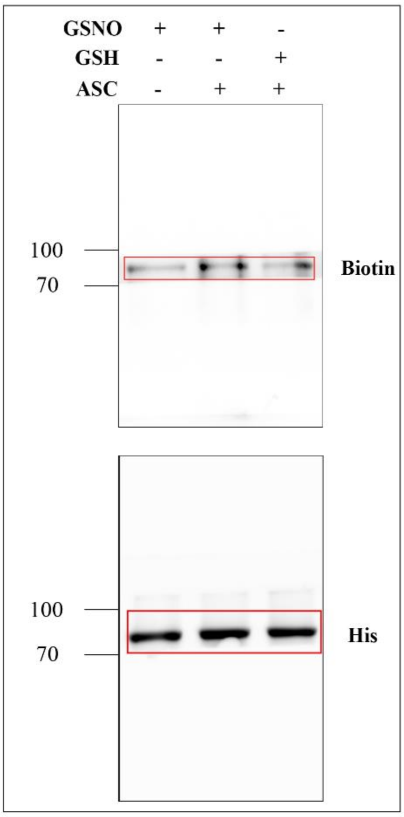

Source data for Figure 6b

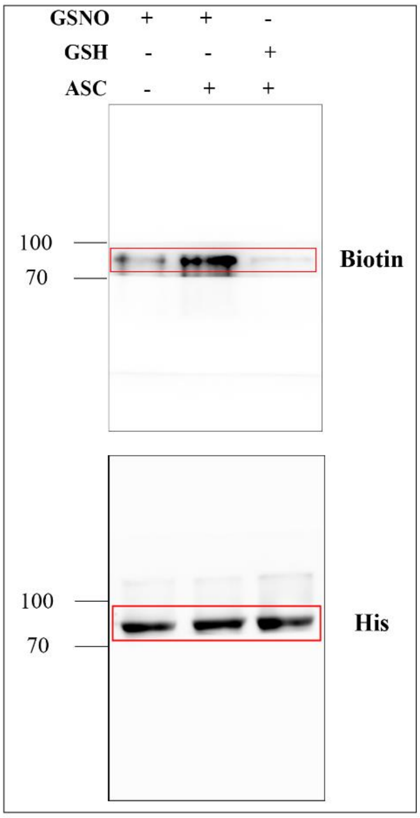

Source data for Figure 6c

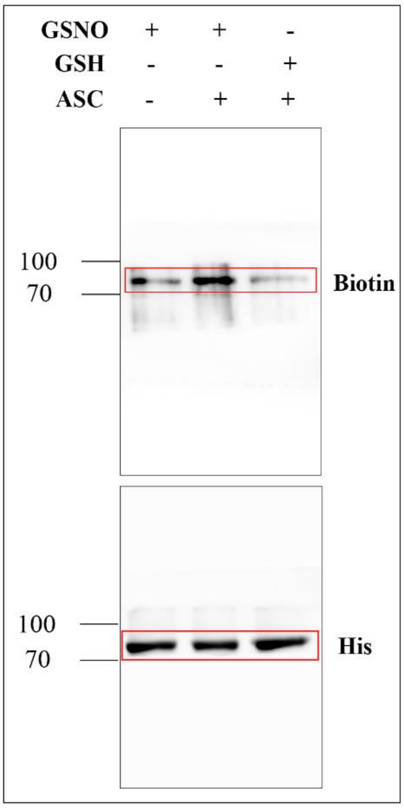

Source data for Figure 6d

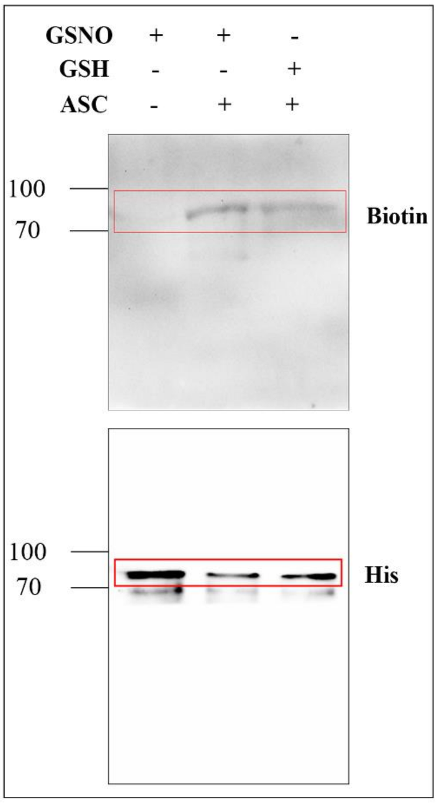

Supplementary Figure 6. Uncropped Western blots.

Source data for Figure 6e

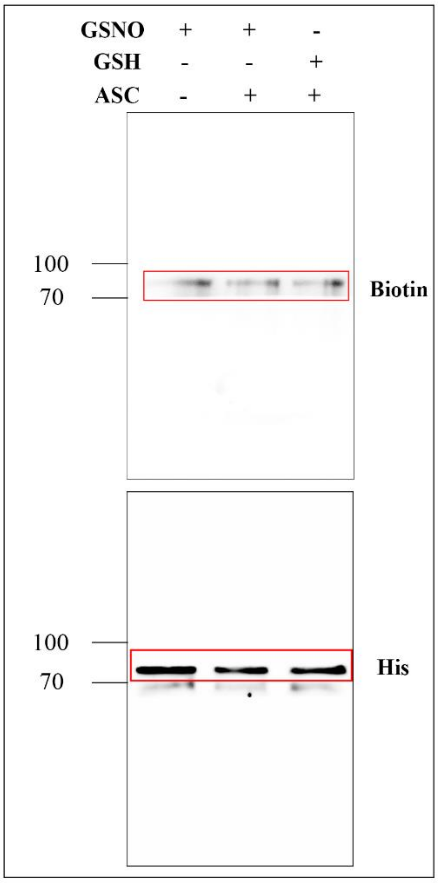

Source data for Figure 6f

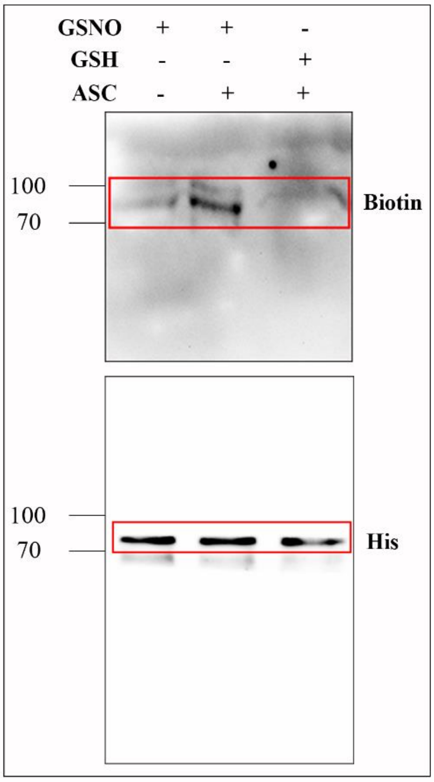

Source data for Figure 6g

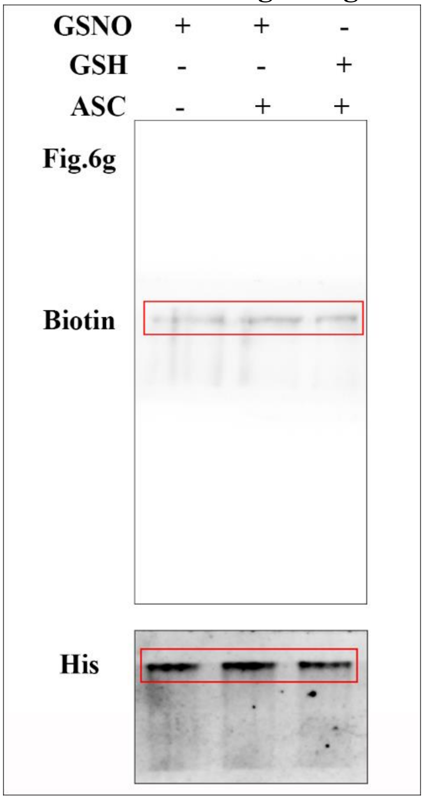

Supplementary Figure 6. Uncropped Western blots.
