# Supplementary material for: GSNOR regulates ganoderic acid content in Ganoderma lucidum under heat stress through S-nitrosylation of catalase
Source: Commun Biol. 2022 Jan 11;5:32. doi: 10.1038/s42003-021-02988-0 (PMC8752759; doi:10.1038/s42003-021-02988-0)
Supplement: Supplementary file 2 — Description of Additional Supplementary Files [file 42003_2021_2988_MOESM2_ESM.pdf]

Description of Additional Supplementary Files

File name: Supplementary Data 1

Description:

1. The source data underlying the graphs in the figure 1a and 1b are shown in Supplementary Data Fig.1.

2. The source data underlying the graphs in the figure 2b -2f are shown in Supplementary Data Fig.2.

3. The source data underlying the graphs in the figure 3b and 3c are shown in Supplementary Data Fig.3.

4. The source data underlying the graphs in the figure 4b and 4c are shown in Supplementary Data Fig.4.

5. The source data underlying the graphs in the figure 6h are shown in Supplementary Data Fig.6h.

6. The source data underlying the graphs in the Supplementary figure S1b are shown in Supplementary Data Fig.S1.

7. The source data underlying the graphs in the Supplementary figure S2b and S2c are shown in Supplementary Data Fig.S2.

8. The source data underlying the graphs in the Supplementary figure S4a-d are shown in Supplementary Data Fig.S4.
